# Single knotless-anchor with two ethicon 2# for Ellman grade III bursal-side partial thickness rotator cuff tears: a cadaveric biomechanical study and short-term clinical evaluation

**DOI:** 10.1186/s12891-023-06841-9

**Published:** 2023-09-01

**Authors:** Ding su-Bao, Baolu- Zhang, Hong- luo, Yang- liu, Rui chen-Li, Yiyuan- Zou, Sheng qiang-Zeng, Shijie- Fu, Gang Liu

**Affiliations:** 1 Department of Orthopedics and Center for Orthopedic Diseases Research, Affiliated Traditional Chinese Medicine Hospital of Southwest Medical University, Lu Zhou, China; 2 Chengdu University of Traditional Chinese Medicine, Chengdu, Sichuan China; 3 Academician Workstation, Guangdong Province Medical 3D Printing Application Transformation Engineering Technology Research Center, Lu Zhou, China; 4 Clinical Base of Affiliated Traditional Chinese Medicine Hospital of Southwest Medical University,, Guangdong Province Medical 3D Printing Application Transformation Engineering Technology Research Center, Lu Zhou, China; 5 School of Nursing, Southwest Medical University, Lu Zhou Sichuan, Lu Zhou Sichuan, China; 6 No.1 Orthopedic hospital of Chengdu,, Chengdu, Sichuan China; 7 Department of Anatomy, Faculty of Medicine, Chiang Mai University, Chiang Mai, Thailand

## Abstract

**Background:**

Several surgical techniques are used to treat bursal-side partial thickness rotator cuff tears (PTRCTs). However, use of single knotless-anchor with two Ethicon 2# repair technique for PTRCTs has not been reported.

**Materials and methods:**

Bursal-side PTRCTs (Ellman grade III, 75% thickness of tears) were created in the supraspinatus tendon in 16 fresh-frozen cadaveric shoulders. The specimens were randomly assigned to two equal groups: (1) Group A (Transtendon repair), a single knotless-anchor repair with two Ethicon 2#; (2) Group B, Conversion repair (Double-row, DR). Post-repair, each specimen was subjected to cyclic loading test from 5 to 100 N (50 cycles), followed by an ultimate failure test. The displacement of greater tuberosity (mm) and ultimate (N) were recorded. In the clinical study, 12 patients diagnosed with Ellman grade III Bursal-side PTRCTs (using a single knotless anchor with two Ethicon 2# repair techniques) were operated on and analyzed. Visual analog scale (VAS), American Shoulder and Elbow Surgeons Score (ASES), Constant-Murley Score (CMS), and range of motion (ROM) were assessed before surgery and at final minimum follow-up (>1year).

**Results:**

There was no significant between-group difference with respect to load-to-failure test (Group A, 359.25 ± 17.91 N; Group B, 374.38 ± 13.75 N, P > 0.05). There were no significant differences with respect to rotator cuff displacement of 10 mm (Group A, 190.50 ± 8.52 N; Group B, 197.25 ± 6.84 N, P > 0.05) and 15 mm (Group A, 282.25 ± 12.20 N; Group B, 291.13 ± 14.74 N, P > 0.05). However, there was significant between-group difference with respect to displacement of 3 and 5 mm (P < 0.05). In the clinical trial, all patients were followed up for an average of 20.4 months (12–29 months). At the last follow-up after surgery(minimum>1year), the VAS score was 0.50 ± 0.67 (0–2), the ASES score was 86.50 ± 3.96 (79–92), the CMS score was 85.08 ± 5.65 (74–93), the mean Forward flexion ROM was 154.00°± 12.48° (131°-169°), and the abduction ROM was 165.00°±13.26° (138°-173°). There was a statistically significant difference between the results of the preoperative and the last postoperative follow-up. The results of the last postoperative follow-up were statistically different from those of the preoperative follow-up (P < 0.05). Regarding complications, stiffness (2 cases) and shoulder impingement (1 case) occurred in 3 cases (25%).

**Conclusion:**

A single knotless anchor with two Ethicon 2# may provide a biomechanically and clinically feasible option for the treatment of bursal-side Ellman grade III PTRCTs, particularly in resource-constrained settings.

**MeSH keywords:**

Bursal-side Ellman Grade III; Single Knotless-anchor; Double-row repair; Biomechanical study; Short-term clinical evaluation.

## Introduction

In the movement of the shoulder, the rotator cuff is crucial. One of a common lesion that results in shoulder pain and dysfunction is partial thickness rotator cuff tears (PTRCTs) [1, 2]. It prevalence in the general population ranges from 13 to 37%, which may be attributed to the high association between age and PTRCT prevalence [3, 4]. In patients aged > 60 years, the reported prevalence is approximately 26% [5]. PTRCTs are frequently classified according to the Ellman method (articular, bursal-side, and intra-tendinous) [6].

Despite their high prevalence, PTRCTs (especially bursal-side tears) have largely been ignored. Most studies focussed on treatment of rotator cuff tears have pertained to full-thickness tears. There are two basic treatment strategies for this lesion: non-operative treatment and operative treatment. Rotator cuff repair is indicated in patients with Ellman grade III PTRCTs [7, 8]. In other types of PTRCTs, surgical intervention is generally indicated in patients who do not respond to nonsurgical treatment for 3–6 months or in younger patients with traumatic injuries [9]. PTRCTs can be treated surgically in a number of ways, including arthroscopic repair and arthroscopic debridement with or without acromioplasty [10, 11]. Conversion repair and in situ repair are two particular repair methods [12, 13].

**Fig. 1:**
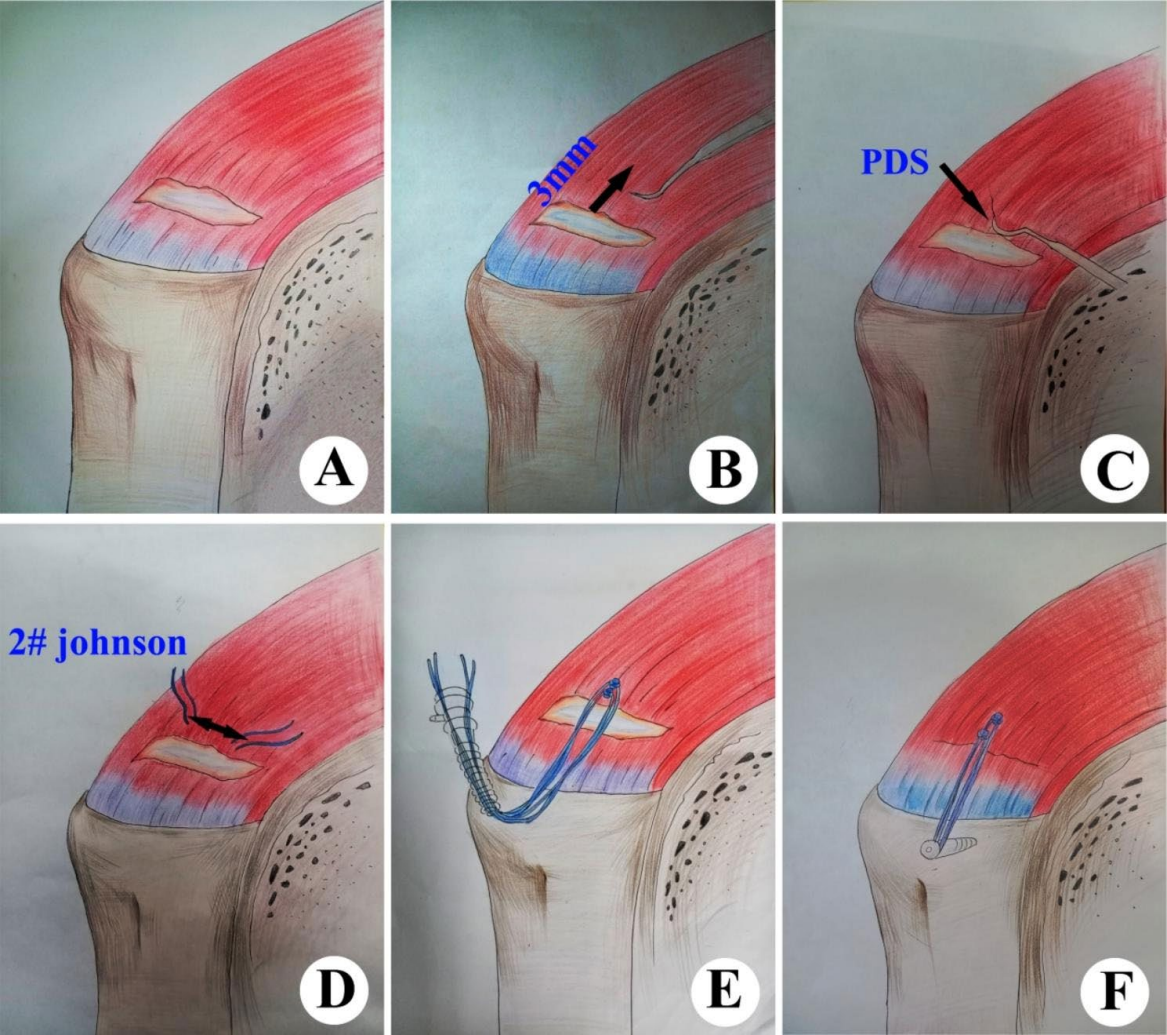
Custom-drawn illustrations of the single knotless-anchor technique. (A) 75% Ellman grade III Bursal-side PTRCTs. **(B)** The distance of the suture hook to puncture. **(C)** PDS thread passing through the hook. **(D)** Ethicon 2# thread passing through the hook. **(E)** The 2 Ethicon 2# thread passing through the Knotless-anchor. **(F)** Fixation of single knotless-anchor

Double-row conversion repair is commonly used to treat mucosal PTRCTs and results in successful clinical and anatomic outcomes with significant improvement in range of motion, strength, and pain relief [14]. However, the high cost of the surgery is a major barrier to its widespread use. In southwestern China, millions of patients are unable to pay the medical costs because of low coverage by health insurance. It is our endeavor to develop a simple and cost-effective alternative treatment for patients who cannot afford this surgery. The purpose of this study was to investigate whether the use of a single knotless anchor with two Ethicon 2# (Johnson, USA, a high polymer polyethylene) for the repair of Ellman grade mucosal PTRCTs III has the same mechanical properties as a double-row repair (Fig. 1).

We hypothesized that the single knotless-anchor with two Ethicon 2# technique may offer a cheap and reliable surgical alternative in resource-constrained settings.

## Materials and methods

### Specimen Selection

This study was approved by the Ethics Committee of the Affiliated Traditional Chinese Medicine Hospital of South-west Medical University. Sixteen fresh-frozen cadaveric shoulder specimens (10 right and 6 left; 9 men and 7 women) were employed in this study. The mean age of donors was 51.20 years (range, 33–65). Written informed consent for use of cadaveric specimens was obtained from the family members of all subjects. The bone density in all specimens was found to be normal (OSTEOCORE-3, Golden, China). The inclusion, criteria were: (i) no history or signs of previous fracture of scapula or clavicle; (ii) normal and full-grown shoulder joint; (iii) no history of shoulder dislocation or operation. The exclusion criteria were: (i) incomplete specimens; (ii) history of drug abuse; (iii) shoulder diseases (e.g. rheumatoid arthritis, tuberculosis of the shoulder joint or RCT).

### Patients selection

**Fig. 2:**
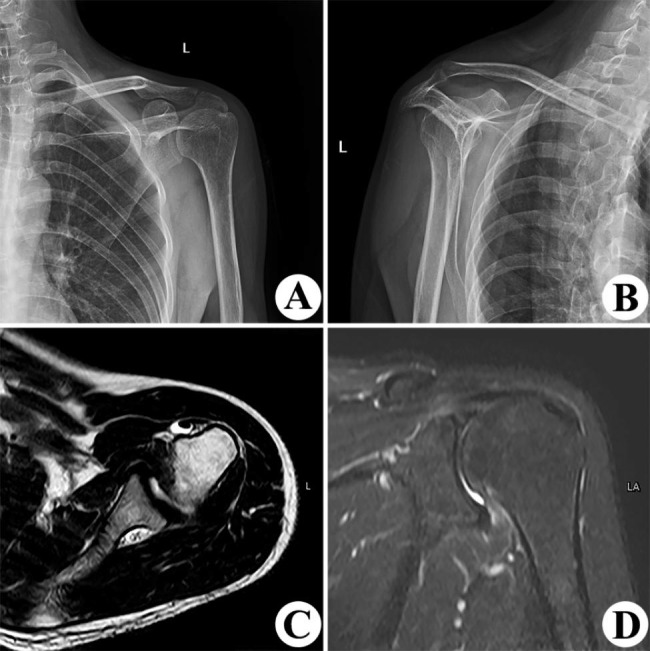
This was a 48-year-old man who presented with recurrent left shoulder pain for more than 2 years diagnosed with bursal-side Ellman grade III PTRCTs. **(A)** Anterior-posterior X-ray of the left shoulder. **(B)** X-ray of the left shoulder in the Y-view. **(C)** MRI of the long head of the biceps tendon **(D)** T2-weighted coronal MRI sequence with bursal-side Ellman grade III PTRCTs.

The inclusion criteria were: (i) Based on the history taken, laboratory findings, radiographic and MRI results, Ellman grade III bursal-side PTRCTs was diagnosed (Fig. 2); (ii) Ultimately, arthroscopy was used to diagnose Ellman grade III bursal-side PTRCTs; (iii) Patients underwent the same surgical procedure and underwent follow-up that lasted more than one year; (iv) Patients and their families signed an informed consent form after being informed about the advantages and disadvantages of the treatment and showing good compliance with follow-up. The exclusion criteria were (i) a good evaluation could not be made because the patient had not returned for follow-up or the follow-up period was shorter than one year; (ii) the patient had a history of epilepsy, mental illness, or drug use; (iii) the patient refused to comply or accept treatment.

### Bursal-side PTRCTs modeling

**Fig. 3:**
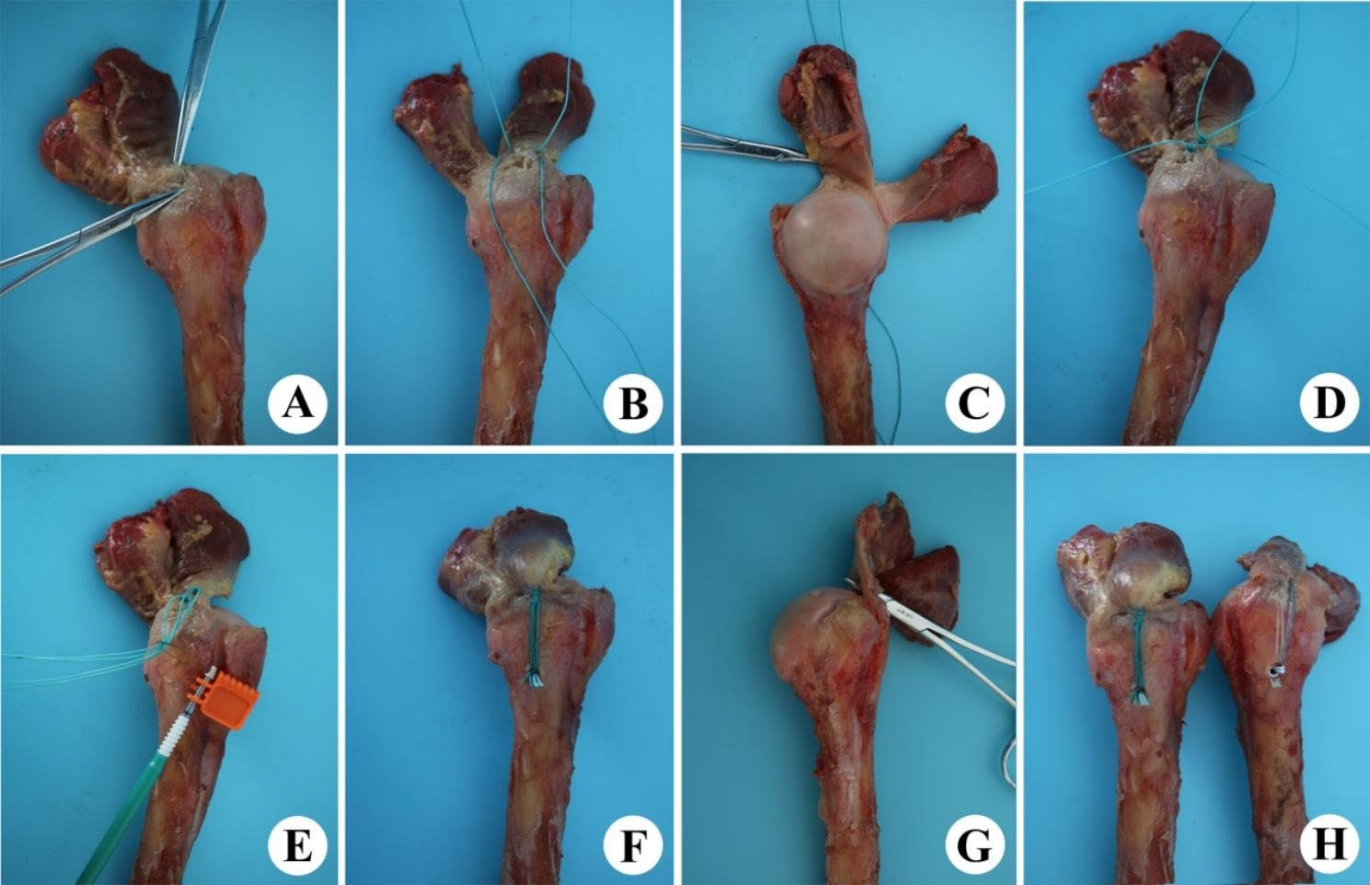
The specific process of the two model repair techniques. **(A)** 75% Ellman grade III PTRCTs created with blade and bone contusion. **(B)** The 2 Ethicon 2# thread suture passing through the supraspinatus. **(C)** The superior capsule and cable are retained. **(D)** The 2 Ethicon 2# thread is knotted. **(E)** The Ethicon 2# passing through the Knotless-anchor while ensuring the location of the lateral row. **(F)** Fixation of the single knotless-anchor. **(G)** The conversion model involved completing PTRCTs to a full-thickness rotator cuff tear. **(H)** Fixation of the conversion repair with double-row technique

All soft tissues of the scapula and humerus were removed from the bone, except for the supraspinatus and infraspinatus tendons. The middle humeral shaft was transected approximately 20 cm from the top of the greater tuberosity. A length of more than 5 cm of the supraspinatus and infraspinatus tendons was preserved at the base of the footprint. The antero-posterior width and thickness of the supraspinatus tendon at the footprint were measured using a digital caliper (Sang-Liang, Japan). We then created 75% bursal-side PTRCTs (Fig. 3A), for which blade repair is usually recommended [15, 16]. To replicate acromion impingement syndrome, we also used a bone contusion (li-Jian, Zhe Jiang, China) to create an impact on this tear (Fig. 3). Subsequently, subjects were randomly assigned to two groups: (1) Group A (transtendon repair), simple knotless anchor repair with two Ethicon 2#; (2) Group B, conversion repair (double row, DR).

### **Model repair technique**

With calipers, all spatial characteristics were meticulously measured (Group A, Fig. 3A-F). In this repair method, a 4.75-mm knotless anchor and 2 Ethicon 2# (Johnson, USA), a high-polymer polyethylene, were employed. A suture hook was used to sew the initial suture at a distance of 3 mm from the upper edge of the injury. The PDS thread was threaded through the hook, and the stitch length was set at two thirds of the injury distance. The hole was made 2 mm above the initial suture for the second suture. Then, 1 to 2 cm distal to the lateral edge of the footprint and parallel to the initial suture’s anchors, a lateral knotless anchor was put in place. All operations were performed by the senior author (Shi Jie-Fu, a shoulder master).

### Conversion repair technique

The conversion repair involved converting the PTRCTs to a full-thickness rotator cuff tear followed by repair (Fig. 3G–H). This repair technique used one 4.5-mm knotless-anchor (Arthrex Inc, Naples, FL, USA) and one suture anchor with Three # 2 (5 metrics) Hi-Fi sutures (ConMed Linvatec, Utica, NY, USA). After conversion of the tear, one suture anchor was used for the medial row and one knotless-anchor was used for the lateral row, using the same techniques as described previously (Fig. 3H) [17]. Finally, we sutured both edges of the supraspinatus by using the 2 Ethicon 5# (Johnson, USA), as illustrated in Fig. 3.

### Load test

**Fig. 4:**
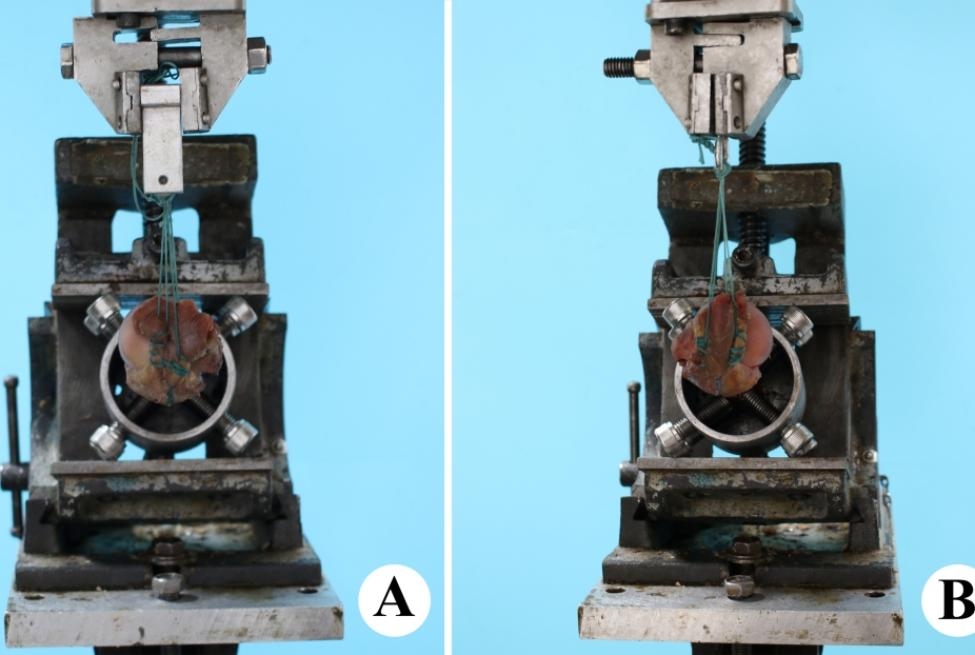
The load test and load-to-failure test. **(A)** Group A, single knotless-anchor repair with two Ethicon 2#. **(B)** Group B, conversion repair with double-row

First, the proximal humerus was fixed with eight screws in a special device (Fig. 4). The supraspinatus was then secured at 90˚ of abduction. The other side of the specimen was secured to the biomechanical testing machine (Bose Electro Force 3520-AT, USA, Fig. 4). Meticulous care was exercised to ensure equal and symmetric tension on the tendon prior to clamping. A small device supplemented with 2 Ethicon 5# was used to secure the supraspinatus tendon. A superior preload of 100 N was then performed to test the specimen fixation stability, time effect and stress relaxation. The electrodynamic testing machine was applied to the load at a constant speed of 5 N/s. Fifty cycles of load test with 5 to 100 N were performed, and the loading interval of each test was kept at 1 min to relieve the stress fatigue. All tests were completed in 12 h at 22° temperature, and the surface of the model was always kept moist with the use of isotonic saline.

### Load to failure test

We tested the ultimate failure load (N) with a constant speed of 1 mm/min in the superior-inferior direction. The mode of failure was recorded for each specimen. The force required to induce displacement of supraspinatus by 3 mm, 5 mm, 10 and 15 mm was recorded. Failure was defined as rupture of supraspinatus, failure of knotless-anchor or suture anchor, or breakage of Ethicon 2#.

### Surgical procedure (clinical part)

**Fig. 5:**
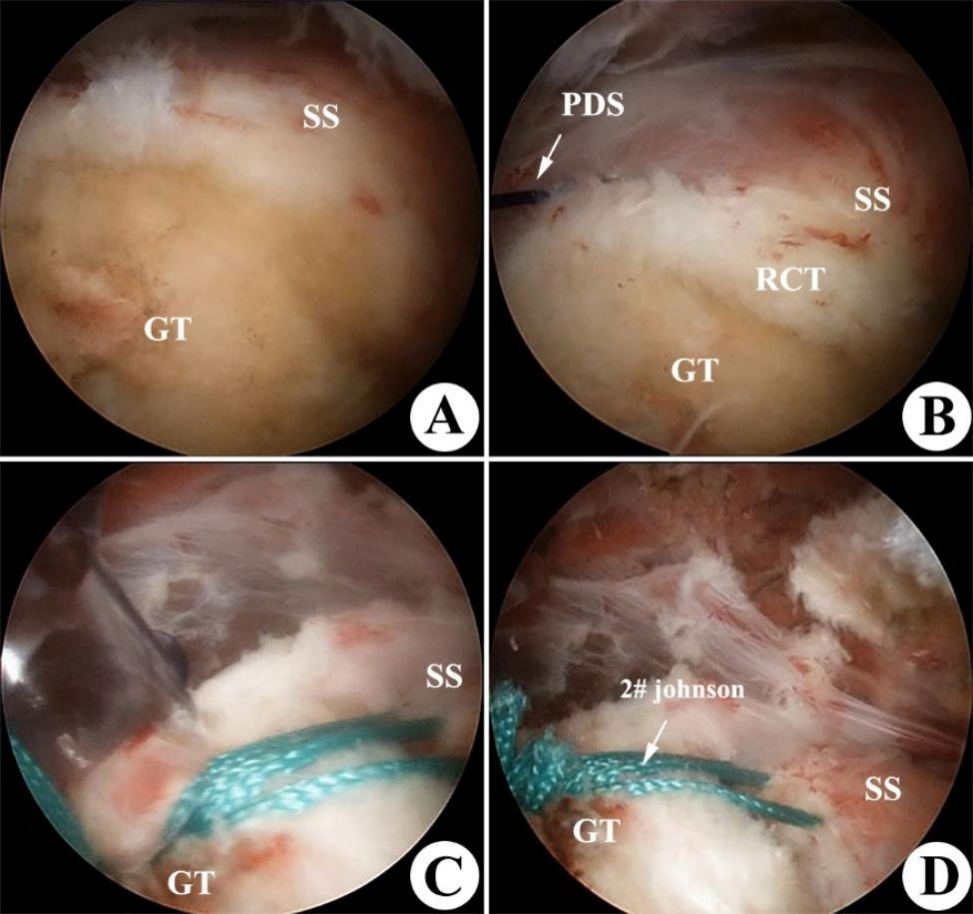
Clinical arthroscopic technique for Ellman grade III PTRCTs. **(A)** Arthroscopic debridement with acromioplasty and assessment for RCT (SS-supraspinatus, GT- greater tuberosity). **(B)** One 2 Ethicon 2 # thread suture is passed through the supraspinatus with the help of PDS, as illustrated in Fig. 5. **(C)** Ethicon 2# is passed through to the Knotless-anchor while ensuring the location of the lateral row under arthroscopic guidance. **(D)** Fixation of Single Knotless-anchor

**Fig. 6:**
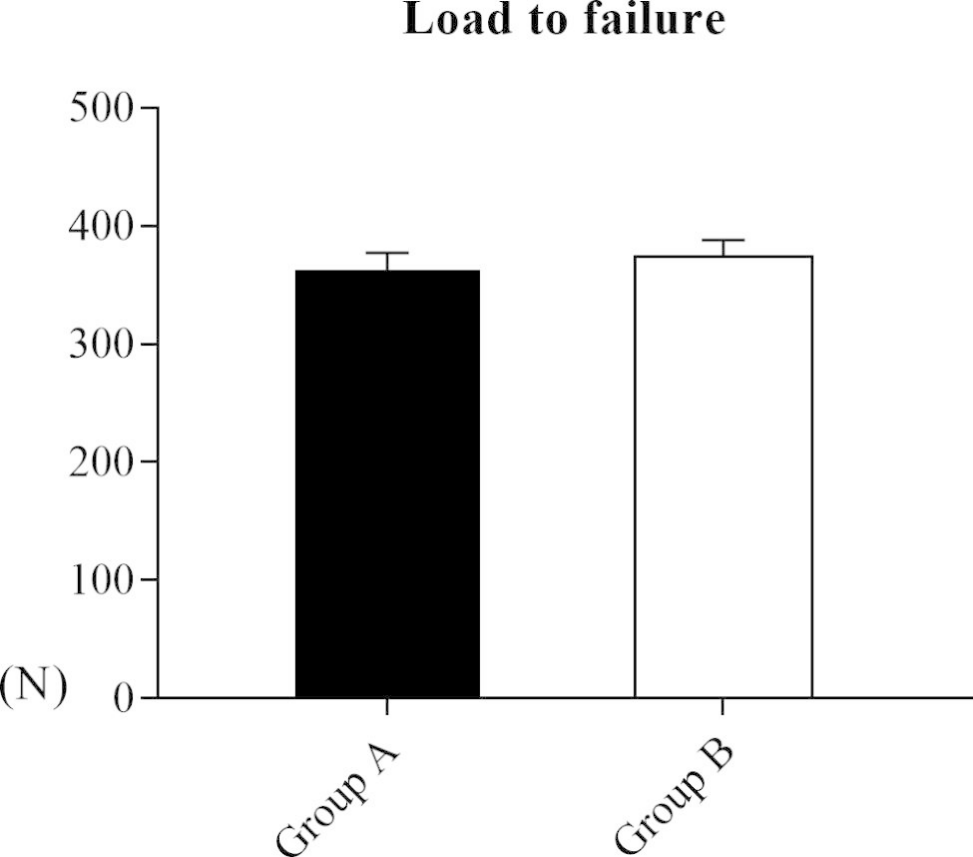
Results of the load-to-failure test. There was no significant difference between the two groups (P > 0.05)

The patient was put under general anesthesia and then placed in a recliner while having his blood pressure gently decreased. The mean arterial pressure was kept at 80 mmHg while the water pump pressure was kept at 60 mmHg. These were the precise actions: (i) Use the posterior technique to access the joint cavity and conduct a complete examination. Pay particular attention to whether the supraspinatus muscle’s footprint was complete and whether the rotator cuff tear’s location was completely exposed (Fig. 5A). (ii) Take into account access from the anterolateral side, pierce the subacromial tissue, remove the hyperemia, edema, and hyperplastic synovial lesion tissue, completely expose the surgical field, assess the condition of the rotator cuff on the side of the bursa, and look at the shape of the acromion. The acromion of the anterolateral edge should be ground down to a thickness of 5–10 mm if subacromial impingement was present in order to increase impingement reduction and increase acromion space. (iii) In order to promote tendon-bone healing, the injured rotator cuff tissue was cleaned with a plane under posterior arthroscopic observation, and the bone surface in the footprint area was refreshed (Fig. 5B). If the tears were larger than 50%, the percentage was then calculated (Ellman grade III). A suture hook was then used to penetrate the skin 3 mm from the upper edge of the tear while operating under arthroscopic supervision. The PDS suture was threaded through the hook, and the stitch length was 2/3 the tear distance. The hole was performed 2 mm above the initial suture for the second suture. A lateral knotless knot is the last anchor was placed 1 to 2 cm (Fig. 6C) distal to the lateral edge of the footprint in line with the anchors of the first suture (as in Fig. 1). (iv) After these operations, the gleno-humeral joint was accessed using an arthroscope to check the area where the rotator cuff footprint was rebuilt. The shoulder was subsequently immobilized with an abduction brace, the wound was irrigated, sutured, and treated before the reconstructed rotator cuff was placed over the footprint region (Fig. 5D).

### Postoperative management

Postoperatively, the shoulder was secured with a shoulder brace in 45º abduction, which decreased postoperative edema, pain, and infection. To lessen swelling, ice packs were also sporadically placed to the affected shoulder. Within two weeks of surgery, simple fist-balling, active and passive elbow flexion, passive abduction, forward flexion, and dorsiflexion of the afflicted shoulder were all possible. Within three weeks following surgery, the afflicted shoulder was gradually moved into pain-free active abduction, forward flexion, and dorsiflexion. After six weeks, the afflicted shoulder began to move through its entire range of motion without experiencing any pain. Imaging was performed as part of routine follow-up exams on the first postoperative day and at months 1, 3, and 6.

### Statistical analysis

Data analysis for performed using Statistical Package for Social Sciences (SPSS) 19.0 (Chicago, IL, USA). All data are presented as mean ± standard deviation. The intra-group homogeneity of variance was assessed using one-way analysis of variance (one-way ANOVA). Between-group differences were assessed using the independent-sample *t*-test (including clinical surgical part). P < 0.05 were considered indicative of statistical significance.

## Results

### Displacement of the supraspinatus

Table 1 summarizes the results at supraspinatus displacements of 3 mm, 5 mm, 10 mm, and 15 mm. Overall, both groups A and B showed a consistent and substantial growing force (P < 0.05). Furthermore, at 10 and 15 mm of supraspinatus displacement, there was no statistically significant difference in force (N) between groups A and B (P > 0.05). At 3 and 5 mm of supraspinatus displacement, there was a significant between-group difference (P < 0.05).

**Table 1:** Displacement of supraspinatus at 3 mm, 5 mm, 10 and 15 mm on load test

Groups	3 mm	5 mm	10 mm	15 mm
A	84.38 ± 6.52^abcd^	130.13 ± 11.12^acd^	190.50 ± 8.52^d^	282.25 ± 12.20
B	100.25 ± 6.39^bcd^	144.25 ± 10.39^ cd^	197.25 ± 6.84^d^	291.13 ± 14.74

Note: a: vs. Group B P<0.05; b: vs. 5 mm P<0.05; c: vs. 10 mm P<0.05; d: vs. 15 mm P<0.05

### Load to failure

Although the conversion repair group showed higher mean strength (N), the between-group difference in this respect was not statistically significant (Group A, 359.25 ± 7.91 N; Group B, 374.38 ± 13.75 N, P > 0.05) (Fig. 6). There were three cases of sutures pulling through the tendon and five cases of loosening or pull-out of the knotless-anchor in group A. However, in Group B, there were seven cases of sutures pulling through the tendon and a case of loosening of knotless-anchor.

### Clinical part

In the clinical trial, 12 patients (5 male and 7 female) aged 36 to 72 years were followed up for an average of 20.4 months (12–29 months). At the last follow-up after surgery(minimum follow-up >1year), the VAS score was 0.50 ± 0.67 (0–2), the ASES score was 86.50 ± 3.96 (79–92), the CMS score was 85.08 ± 5.65 (74–93), the mean forward flexion ROM was 154.00°± 12.48° (131°-169°), and the abduction ROM was 165.00°±13.26° (138°-173°). The detailed results can be found in Table 2. There was a statistically significant difference between the results of the preoperative and the last postoperative follow-up. The results of the last postoperative follow-up were statistically significantly different from those of the preoperative follow-up (P < 0.05). Regarding complications, stiffness (2 cases) and shoulder impingement (1 case) occurred in 3 cases (25%).

**Table 2:** Comparison of preoperative and postoperative (minimum follow-up >1year) VAS, ASES, CMS, forward flexion, abduction in clinical patients

Item (groups)	VAS (score)	ASES (score)	CMS (score)	Forward flexion (degree)	Abduction (degree)
Preoperative	3.80 ± 1.14	37.20 ± 9.25	42.30 ± 8.193	72.70 ± 12.75	67.80 ± 14.70
Postoperative	0.50 ± 0.67^a^	86.50 ± 3.966^a^	85.08 ± 5.648^a^	154.00 ± 12.483^a^	165.00 ± 13.26^a^

Note: a: vs. Group B P<0.05

## Discussion

Our study demonstrate single knotless-anchor and double-row techniques have a similar biomechanical effect. We observed no significant difference between group A and B with respect to the 10 and 15 mm supraspinatus tendon displacement and the results of load to failure test. This indicates that a single knotless-anchor is a viable technique for treatment of bursal-side Ellman grade III PTRCTs. In addition, this technique worked well to restore shoulder joint function after a short-term clinical follow-up.

DR technique is the most commonly performed surgery for rotator cuff repair in clinical settings. Studies have shown that DR rotator cuff repair confers superior biomechanical properties compared to single-row repair (SR). Moreover, the DR technique has been shown to reconstruct the anatomic footprint of the rotator cuff significantly better than SR repair [18, 19]. In our study, there was no significant between-group difference with respect to the results of load to failure test. This indicates that the single knotless-anchor with 2 Ethicon 2# can also reconstruct the anatomic footprint and confer biomechanical properties similar to that after DR rotator cuff repair. Moreover, regarding the 10 and 15 mm displacement of the supraspinatus, our results demonstrate that a single knotless-anchor can withstand the horizontal biomechanical traction to achieve a balance. On the other hand, concerning the supraspinatus displacement on 3 and 5 mm, the DR group showed superior biomechanical properties. This finding suggested that the suture anchor in the medial-row provided firm power and reduced the micro-motion of the medial rotator cuff.

Although the single knotless-anchor for Bursal-side Ellman grade III PTRCTs with two Ethicon 2# is a simple, cheap and effective technique, the essence of our method is the in situ repair (transtendon repair). In the study by Gonzalez-Lomas, the ultimate failure load after transtendon repair was significantly higher than that after conversion repair with DR [20]. In another primary research, transtendon repair showed a higher ultimate failure load than double-row repair following full-thickness conversion [21]. These findings are consistent with our findings, which showed the biomechanical advantages of tendon repair. Moreover, clinically, transtendon repair has been shown to be more effective than conversion to a full-thickness tear with subsequent repair. Hytham Salem demonstrated that the in situ repair technique with double-row suture anchors for repair of bursal-sided PTRCTs provided a better fixation of the rotator cuff tissue while preserving the anatomy of the medial footprint [22]. This particular study was one of the reasons that inspired the present study. Our technique provides a simple, cheap and effective alternative for repair of bursal-side Ellman grade III PTRCTs.

There are some considerations for the use of this technique. This revealed that use of 2 Ethicon 2# sutures is associated with a higher risk of cutting supraspinatus. Furthermore, theoretically, this might increase the failure of fixation or re-rupture rates. On the other hand, Ethicon 2# sutures may cover weak areas in the tendon and decrease the load to failure test. Therefore, the failure test does not simulate the natural failure biomechanics. Therefore, the following surgical indications should be mandatory for this methods: (i) confirmation of good elasticity or quality of the supraspinatus after arthroscopic debridement; (ii) normal bone density of the lateral row of the greater tuberosity; (iii) age < 60 years.

In our short-term clinical evaluation, this method proved effective in restoring shoulder joint function. At the last follow-up after surgery, the ASES score was 86.50 ± 3.96 (79–92), the CMS score was 85.08 ± 5.65 (74–93), the mean forward flexion ROM was 154.00°± 12.48° (131°-169°), and the abduction ROM was 165.00°±13.26° (138°-173°). Compared with the classic technique of partial rotator cuff repair (with arthroscopic conversion to full-wall tears), the mean ASES and Constant scores reported by Kim improved significantly to 90.80 and 83.00, respectively, at postoperative follow-up of bursal-side PTRCTs fixed with the suture bridge technique [23], and similar clinical results were obtained with our technique. Meanwhile, Nuri Aydin reported that arthroscopic repair of high-grade bursal-side PTRCTs after conversion to full-thickness ruptures is a reliable surgical technique with good functional results (mean CMS score improved from 38.9 preoperatively to 89.2 and 87.8 2 years and 5 years after surgery, respectively) and pain was relieved at both mid- and long-term follow-up [24]. These are further evidence of the reliability of our method. In addition, Planche reported in a comprehensive review that after surgical repair of partial rotator cuff tears using various surgical techniques, mean ASES scores of the shoulder at the end of postoperative follow-up ranged from 76.1 to 85.1 [25].

Listed below are some benefits of our approach: (i) A repair that solely addresses the bursal layer can produce the same clinical results or structural integrity as a repair that addresses all layers [26]. (ii). Moreover, we keep the cable, which is the supraspinatus’ main load-bearing component [27]. For Ellman grade III PTRCTs on the bursal side, the single knotless anchor is a practical substitute, especially in environments with limited resources. (iv) The larger tuberosity’s medial row osteoporosis does not prevent this surgery from being performed.

However, it is important to highlight some of our study’s limitations. First off, because there weren’t enough human samples available, this study only employed 16 fresh-frozen samples. In a later investigation, we’ll use more cadaveric shoulders. Second, not all fixation methods, such as a typical suture bridge, were used in our investigation due to the scarcity of cadaver specimens. Thirdly, there is a need for a future clinical comparison study comparing our technique and conversion to a full-thickness tears technique at this time, we only included a small number of clinical cases (12 cases).

## Conclusion

A single knotless anchor with two Ethicon 2# may provide a biomechanically and clinically feasible option for the treatment of bursal-side Ellman grade III PTRCTs, particularly in resource-constrained settings.

## Data Availability

All of the data in this study are obtained from experiments. The data used and analysed in this study are available from the corresponding author on reasonable request.

## Declarations

### Statement

All the methods and procedures carried out in this study were in accordance with relevant guidelines and regulation (Declaration of Helsinki).
